# United States Pooled Cohort Cardiovascular Disease Risk Scores in Adults With Diabetes Mellitus

**DOI:** 10.1016/j.jacadv.2024.101448

**Published:** 2024-12-13

**Authors:** Yanglu Zhao, Ralph B. D’Agostino, Shaista Malik, Karol E. Watson, Alain G. Bertoni, Matthew J. Budoff, Loretta Cain, Adolfo Correa, Aaron R. Folsom, David R. Jacobs, Elizabeth Selvin, Nathan D. Wong

**Affiliations:** a Department of Epidemiology, University of California, Los Angeles, California, USA; b Mary and Steve Wen Cardiovascular Division, Department of Medicine, University of California-Irvine, Irvine, California, USA; c Department of Biostatistics, Boston University, Boston, Massachusetts, USA; d Department of Epidemiology and Prevention, Wake Forest School of Medicine, Winston-Salem, North Carolina, USA; e Division of Cardiology, Lundquist Institute, Torrance, California, USA; f Department of Medicine, University of Mississippi Medical Center, Jackson, Mississippi, USA; g Division of Epidemiology and Community Health, School of Public Health, University of Minnesota, Minneapolis, Minnesota, USA; h Department of Epidemiology, John Hopkins Bloomberg School of Public Health, Baltimore, Maryland, USA

**Keywords:** cardiovascular disease, diabetes mellitus, risk factors, risk prediction, risk scores

## Abstract

**Background:**

There is significant heterogeneity in cardiovascular disease (CVD) risk among patients with diabetes mellitus (DM).

**Objectives:**

The purpose of this study was to develop risk scores for total CVD and its components from a contemporary pooled, observational cohort of U.S. adults with DM.

**Methods:**

CVD-free adults with DM aged 40 to 79 years were pooled from 4 U.S. population-based cohorts (CARDIA [Coronary Artery Risk Development in Young Adults], Framingham Offspring, Jackson Heart Study, and the MESA (Multiethnic Study of Atherosclerosis) studied since 2000. Baseline DM-specific and non-DM–specific CVD risk factors were evaluated as predictors. We developed 10-year DM Risk Scores (DMRS) for total CVD, atherosclerotic CVD (ASCVD), coronary heart disease (CHD), heart failure (HF) and stroke. Score performance was validated internally and externally.

**Results:**

We included 2,174 adults with DM mean age 59.2 ± 10.5 years, 55.4% female and 47.5% Black followed up to 10 years. Age, sex, HbA1c, creatinine, systolic blood pressure, DM medication, and smoking were the most important predictors. The DMRS had good internal discrimination (c-statistics 0.72, 0.72, 0.72, 0.79 and 0.73 for CVD, ASCVD, CHD, HF, and stroke) and calibration (calibration slopes 0.93, 0.95, 0.93, 0.98, and 0.89 for CVD, ASCVD, CHD, HF, and stroke; Greenwood Nam-D’Agostino calibration tests were significant for CHD (*P* < 0.01) and CVD (*P* < 0.05) but not for ASCVD, HF, and stroke). From external validation in 2 other cohorts, the DMRS outperformed current risk scores.

**Conclusions:**

Our U.S. pooled cohort DMRS for predicting CVD events demonstrated good predictive performance for assessing CVD risk in adults with DM.

Diabetes mellitus (DM) has been designated as a risk equivalent for coronary heart disease (CHD) events.^1^^,^^2^ However, recent studies show DM to have wide heterogeneity in CHD risk, indicating all DM patients are not “CHD risk equivalents” and suggesting the need for further risk stratification.^3^, ^4^, ^5^, ^6^

Current cardiovascular disease (CVD) risk assessment for persons with DM is limited to risk scores derived from the general population, such as the Framingham Risk Score (FRS) or the Pooled Cohort Equation (PCE) for atherosclerotic cardiovascular disease (ASCVD),^7^^,^^8^ as well as the recently released PREVENT risk scores for CVD, ASCVD, and heart failure (HF),^9^ or are from other countries or regions, such as the UKPDS (U.K. Prospective Diabetes Study) risk engine ^10^ and SCORE-2 Diabetes from Europe.^11^ While the PCE ^8^ treats DM as a binary factor that does not address its heterogeneity in risk nor include other DM specific factors, the recent PREVENT risk score ^9^ requires body mass index and estimated glomerular filtration rate (eGFR) and has options for including glycated hemoglobin (HbA1c) and urine albumin creatinine ratio (UACR) to further refine risk and the SCORE-2 Diabetes algorithm from Europe does add diabetes-specific factors including HbA1c, duration of diabetes, and chronic kidney disease.^11^ Other risk scores have inadequate calibration or discrimination in external validation, with a tendency to overestimate the risk in modern populations which can lead to overtreatment.^12^^,^^13^

We aimed to develop a set of pooled cohort DM risk scores (DMRS) for total CVD, ASCVD and individually for CHD, stroke, and HF from U.S. subjects with DM among 4 U.S. cohorts, each having over 10 years of follow-up and being ethnically diverse overall. The DMRS were then externally validated in 2 DM cohorts.

## Methods

### Study participants

Development cohort: We pooled 4 U.S. prospective cohorts with diverse ethnic and geographic backgrounds: CARDIA (Coronary Artery Risk Development in Young Adults Study), Framingham Heart Study Offspring cohort (FHS Offspring), JHS (Jackson Heart Study) (excluding participants already in the ARIC (Atherosclerosis Risk In Communities Study), and the MESA (Multi-Ethnic Study of Atherosclerosis).^14^, ^15^, ^16^, ^17^ We included subjects aged 40 to 79 years with DM and free of known CVD at baseline. DM was defined as 1) physician diagnosed DM; 2) use of insulin or oral diabetes medication; 3) a fasting blood glucose level of ≥126 mg/dL; 4) a nonfasting blood glucose level or 2-hour oral glucose tolerance test ≥200 mg/dL; and/or (5) a glycated hemoglobin (HbA1c) ≥6.5% at the time of (or earlier than) the identified baseline visit where HbA1c and other risk factor information were available (2005 in CARDIA, 1998-2001 for FHS Offspring, 2000-2002 in JHS, and 2003-2004 in MESA). Participants were excluded if they had a history of CVD at baseline.

Validation cohort: We used 2 external validation cohorts to test the performance of the DMRS. The first included ARIC,^18^ a multicentered, prospective observational study investigating the causes of atherosclerosis and its clinical outcomes. We used as baseline the 2nd ARIC exam in 1991 to 1992 where HbA1c measures were available. The second validation cohort was a subgroup of CVD-free participants from the ACCORD (Action to Control Cardiovascular Risk in Diabetes) Follow On (ACCORDION) cohort.^19^ The ACCORD trial examined whether intensive versus standard hypoglycemic treatment would reduce CVD risk in people with type 2 DM. We included participants who were assigned to usual care for glucose, lipids, and blood pressure but also conducted a sensitivity analysis in those with all treatment combinations.

### Follow-up and endpoints ascertainment

We defined incident CVD as myocardial infarction (MI), cardiac revascularization, stroke, HF, or CVD death. Incident ASCVD was defined as MI, stroke, or CVD death. Incident CHD included MI, cardiac revascularization, or CHD death. The adjudication process for events involved a panel to review hospitalization and death data per study protocols previously published.^14^, ^15^, ^16^, ^17^, ^18^, ^19^ We did not include peripheral arterial disease as an endpoint because of it not being adjudicated in the JHS. All events were adjudicated from medical records and death certificates by the endpoint committees from each of the studies. For better estimation of the effect of predictors and the 10-year baseline risk (*S*_*10*_), follow-up time for each cohort was truncated at 10 years.

A summary of the DMRS development is shown in the Central Illustration.

**Central Illustration:**
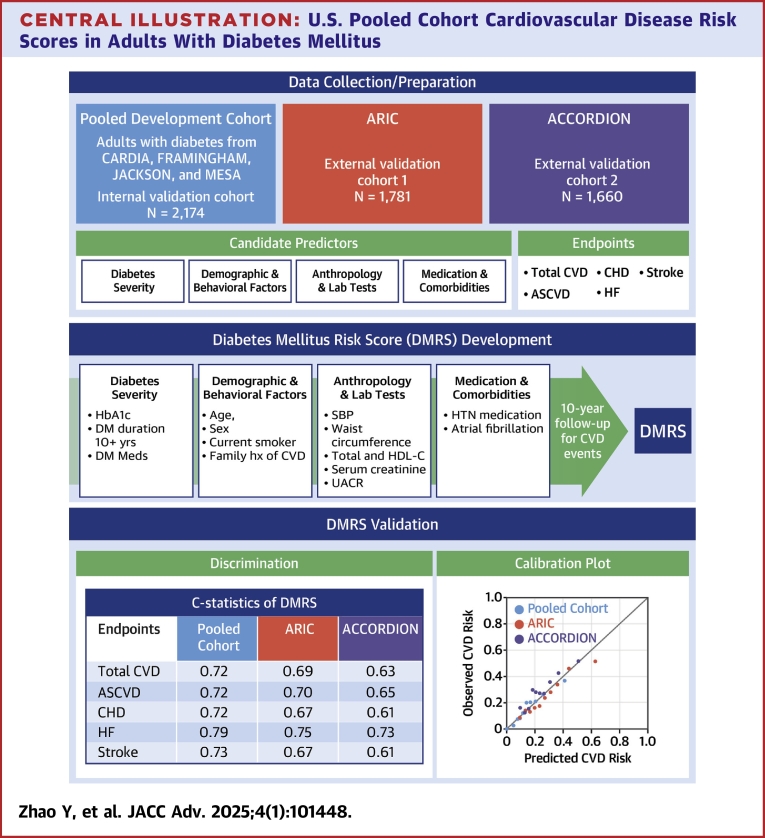
U.S. Pooled Cohort Cardiovascular Disease Risk Scores in Adults With Diabetes Mellitus ASCVD = atherosclerotic cardiovascular disease; CHD = coronary heart disease; CVD = cardiovascular disease; DM = diabetes mellitus; HF = heart failure; HDL-C = high-density lipoprotein-cholesterol; SBP = systolic blood pressure; UACR = urine albumin creatinine ratio.

### Statistical analysis

Continuous data are presented as mean ± SD, as well as median with 25th-75th percentiles if skewed. Categorical data are presented as count (percentage). Risk factor candidates for our scores collected at baseline included age, sex, education level, and typically available office visit information including smoking status, alcohol consumption, family history of premature CVD (age <55 years for father or <60 years for mother), systolic and diastolic blood pressure (SBP/DBP), heart rate, atrial fibrillation, DM duration, body mass index, waist circumference, total cholesterol, high density lipoprotein cholesterol (HDL-C), triglycerides, fasting glucose, HbA1c, UACR, and serum creatinine. Lipid-lowering medication, hypertension medication, and hypoglycemic medication were also collected. Missing values of baseline risk factors were filled using multiple imputation with fully conditional specification methods. We first used elastic net regularization for survival data to reduce the number of correlated risk factors.^20^ Then, the remaining risk factors were examined in the full model. Risk factors with *P* < 0.15 in the full model remained in the final model, with age, sex, and race being forced in the model. Proportional hazards assumptions were checked using interactions of the predictors and a function of survival time included in the model.

Fine-Gray subdistribution hazard models using all-cause mortality as a competing risk with the selected risk factors produced both relative risks as HRs and an estimation of the absolute risks of an event occurring at year 10 with 95% CIs. An individual’s estimated absolute DMRS was calculated as:

$$R=1-{\left(S_{10}\right)}^{e^{\left(\Sigma \;beta*X_{Individual}-Beta*X_{Mean}\right)}}$$

where S_10_ is the population mean survival for individual event at year 10, beta is coefficient of each risk factor for individual event, X_individual_ is the individual value of risk factor X, and X_mean_ is the population mean of risk factor X.

Internal validation was done using the 200 bootstraping samples with replacement. External validation was done in ARIC and ACCORDION separately. We compared performance regarding discrimination and calibration between the above DMRS and existing risk scores for CVD [compared to FRS for total CVD and PREVENT for CVD],^7^^,^^9^ ASCVD (compared to PCE for ASCVD and PREVENT for ASCVD),^8^^,^^9^ CHD (compared to FRS and UKPDS for CHD),^10^^,^^21^ HF (compared to FRS for HF and PREVENT for HF),^22^ and stroke (compared to UKPDS for stroke).^23^ We used Harrell’s C-statistics to examine the discrimination and Greenwood Nam-D’Agostino test for calibration. Given baseline of ARIC was about 10 years earlier than the pooled development cohort, we did recalibration before testing to accommodate cohort effect.

Statistical analysis was done using R Version 3.5.3 and SAS version 9.4 (SAS Institute Inc). A 2-sided *P* value < 0.05 was considered statistically significant unless otherwise noted.

## Results

Our pooled derivation cohort included 2,174 adults with DM with mean age 59.2 + 10.5 years (55.4% female and 47.5% Black). Baseline characteristics and events are shown in Table 1 and Supplemental Table 1 by development cohort. Over 10 years of follow-up, 339 total CVD, 197 ASCVD, 183 CHD, 123 HF, and 87 stroke incident events occurred in the pooled cohort.

**Table 1:** Characteristics of the Pooled Cohort

	Pooled Development Cohort (n = 2,174)	Validation Cohort 1 ARIC (n = 1,781)	Validation Cohort 2- ACCORDION (n = 1,660)
Age, y	59.2 ± 10.5	57.7 ± 5.7	63 ± 6
Female	970 (44.6%)	819 (46%)	913 (55%)
Race groups			
White	743 (34.2%)	1,049 (58.9%)	1,005 (60.5%)
Black	1,032 (47.5%)	729 (40.9%)	346 (20.8%)
Other races	399 (18.4%)	3 (0.2%)	309 (18.6%)
Above high school education	1,277 (58.7%)	668 (37.5%)	982 (59.2%)
Current smoking	291 (13.4%)	354 (19.9%)	227 (13.7%)
Alcohol consumption	1,000 (46%)	790 (44.4%)	1703 (102.6%)
Family history of CVD	707 (32.5%)	986 (55.4%)	731 (44%)
Systolic blood pressure, mm Hg	128.8 ± 19.7	128.1 ± 19.6	136.7 ± 16.3
Diastolic blood pressure, mm Hg	73.5 ± 10.4	73.2 ± 10.5	75.7 ± 10
Body mass index, kg/m^2^	32.5 ± 7.4	30.9 ± 6	32.2 ± 5.4
Waist circumference, cm	106.2 ± 16	105.9 ± 14.5	106.2 ± 13.7
Total cholesterol, mg/dL	189.1 ± 39.5	212.4 ± 42.8	185.5 ± 39.5
HDL-C, mg/dL	47.8 ± 14.3	44.3 ± 14.4	43.2 ± 11.5
	45 (38-55)	42 (34-52)	41 (35-49)
LDL-C, mg/dL	111.9 ± 35.2	135.5 ± 38.5	106.7 ± 32.7
Triglycerides, mg/dL	153.7 ± 113.9	168.2 ± 134.2	180.3 ± 112
	126 (87-183)	138 (99-197)	154 (104-218)
Serum creatinine, mg/dL	0.9 ± 0.4	1.2 ± 0.5	0.9 ± 0.2
	0.9 (0.7-1.0)	1.1 (1-1.3)	0.9 (0.7-1)
UACR, mg/g	86.4 ± 23.4	62.5 ± 13	78.6 ± 233.3
			13 (6-41)
HbA1c, %	7.2 ± 1.7	7.4 ± 2.1	8.2 ± 1.0
Fasting glucose, mg/dL	140.1 ± 53.1	171.6 ± 77.7	172.7 ± 52.3
	127 (105-159)	141 (126-194)	167 (138-202)
DM onset age, y	53.2 ± 12.9	53.2 ± 8.9	52.7 ± 8.6
Heart rate, beats/min	70.5 ± 12.3	69.3 ± 11.3	70.6 ± 11.0
Atrial fibrillation	16 (0.7%)	3 (0.2%)	20 (1.2%)
Lipid-lowering medication	614 (28.2%)	130 (7.3%)	935 (56.3%)
Hypertension medication	1,338 (61.5%)	856 (48.1%)	1,322 (79.6%)
Hypoglycemic medication	1,264 (58.1%)	569 (31.9%)	1,563 (94.2%)
Events during 10-y follow-up			
Incident CVD	339 (15.6%)	449 (25.2%)	428 (25.8%)
Incident ASCVD	197 (9.1%)	282 (15.8%)	223 (13.4%)
Incident CHD	183 (8.4%)	272 (15.3%)	226 (13.6%)
Incident HF	123 (5.7%)	214 (12%)	72 (4.3%)
Incident Stroke	87 (4%)	114 (6.4%)	60 (3.6%)

Values are mean ± SD, n (%), or median (Q1-Q3).

ASCVD = atherosclerotic cardiovascular disease; CHD = coronary heart disease; CVD = cardiovascular disease; eGFR = estimated glomerular filtration rate; HbA1c = hemoglobin A1c; HDL-C = high density lipoprotein-cholesterol; HF = heart failure; LDL-C = low density lipoprotein-cholesterol; UACR = urine albumin/creatinine ratio.

### Risk prediction model development

Table 2 shows the Fine-Gray models used to estimate the absolute 10-year risks. For the 10-year CVD risk, age, sex, current smoking status, family history of CVD, SBP, HbA1c, waist circumference, total cholesterol, HDL-C, UACR, serum creatinine, diabetes duration over 10 years, atrial fibrillation, hypertension medication, and DM medication were included in the final model. We ranked predictors in each score according to their chi-square contribution and found age, sex, HbA1c, serum creatinine, SBP, DM medication, and current smoking appeared most frequently in the first half of strongest predictors. In the pooled cohort, the average predicted 10-year risks were 15.6%, 9.1%, 8.5%, 5.8%, and 4.2% for CVD, ASCVD, CHD, HF, and stroke, respectively.

**Table 2:** Diabetes Mellitus Risk Score for Cardiovascular Disease and Its Components

	β Coefficients				
	CVD	ASCVD	CHD	HF	Stroke
Age, per 1 y	0.0329	0.0218	0.0397	0.0589	0.0095
Male	0.3057	0.3757	0.5611	0.0988	0.4065
Current smoker	0.5105	0.5309	0.5828	0.3624	/
Family history of CVD	/	0.4563	/	/	0.6234
SBP, per 1 mm Hg	0.0085	0.0102	/	/	0.0172
Waist circumference, per 1 mm	0.0724	0.0784	0.0717	0.0790	0.1204
HbA1c, per 1%	/	−0.0122	/	0.0133	−0.0132
Total cholesterol, per 1 mg/dL	0.0032	0.0036	0.0039	0.0043	0.0038
Ln (HDL-C), per 1 U	−0.4683	−0.7223	−0.5694	/	−0.8655
Ln (UACR)	0.0925	0.1231	/	0.2185	0.1135
Ln (serum creatinine), per 1 U	0.5995	0.5944	0.5784	0.6655	/
DM duration over 10 y	0.2432	/	0.3597	/	/
Taking medication for HTN	0.2302	/	0.0000	0.3493	/
Taking medication for DM	0.4187	0.4446	0.4524	0.5031	0.4807
Atrial fibrillation	0.8871	0.9840	/	1.1911	1.3694
Other parameters^a^					
Σβ∗X_Mean_	3.1897	0.6677	2.0231	7.3714	0.6049
S_10_	0.8730	0.9287	0.9330	0.9624	0.9692
Summary of 10-y risk score (%)					
Mean (95% CI)	15.6 (15.2-16.1)	9.1 (8.8-9.4)	8.5 (8.2-8.8)	5.8 (5.5-6.1)	4.2 (4.0-4.3)

“/” means the component is not part of the risk score equation.

ASCVD = atherosclerotic cardiovascular disease; CHD = coronary heart disease; CVD = cardiovascular disease; HbA1c = hemoglobin A1c; HDL-C = high density lipoprotein-cholesterol; HF = heart failure; SBP = systolic blood pressure; UACR = urine albumin/creatinine ratio.

a 10-year event risk is calculated as: $R=1-{\left(S_{10}\right)}^{e^{\left(\Sigma \;beta*X-Beta*X_{Mean}\right)}}$, where Σbeta∗X is the sum of beta coefficient∗individual’s predictor values.

### Internal validation

The internal performance of the DMRS in bootstrapped samples were examined overall and by sex. The Harrell’s C-statistic was 0.72 (95% CI: 0.70-0.75) for the DMRS-CVD [0.69 (95% CI: 0.65-0.73) for male and 0.73 (95% CI: 0.70-0.77) for female]. Internally the C-statistics demonstrated good to excellent discrimination and calibration: HF risk score showed the best discrimination ability with C-statistics of 0.79 (95% CI: 0.75-0.82) overall [0.77 (95% CI: 0.71-0.82) for male and 0.80 (95% CI: 0.74-0.85) for female]. Internal discrimination was generally better in females than males (Table 3, Central Illustration).

**Table 3:** Internal Validation of the Diabetes Mellitus Risk Score

	Total	Male	Female
CVD			
Harrell’s C-statistics (95% CI)	0.72 (0.70-0.75)	0.69 (0.65-0.73)	0.73 (0.70-0.77)
Calibration (slope/intercept/chi2)	0.93/0.015/17.18^a^	0.91/0.017/7.11	1.08/−0.01/22.36^c^
ASCVD			
Harrell’s C-statistics (95% CI)	0.72 (0.68-0.75)	0.68 (0.63-0.73)	0.73 (0.68-0.77)
Calibration (slope/intercept/chi2)	0.95/0.004/7.06	0.86/0.017/12.78	0.99/0/6.12
CHD			
Harrell’s C-statistics (95% CI)	0.72 (0.68-0.75)	0.68 (0.64-0.72)	0.7 (0.64-0.75)
Calibration (slope/intercept/chi2)	0.93/0.006/21.84^b^	0.75/0.039/6.86	1.03/−0.002/10.45^a^
HF			
Harrell’s C-statistics (95% CI)	0.79 (0.75-0.82)	0.77 (0.71-0.82)	0.8 (0.74-0.85)
Calibration (slope/intercept/chi2)	0.98/0.005/9.36	0.78/0.022/10.46	1.13/−0.006/4.08
Stroke			
Harrell’s C-statistics (95% CI)	0.73 (0.68-0.78)	0.71 (0.64-0.79)	0.73 (0.65-0.8)
Calibration (slope/intercept/chi2)	0.89/0.005/2.67	0.95/0.002/0.06	0.96/0.002/1.7

Internal validation was conducted in 200 bootstrap samples.

ASCVD = atherosclerotic cardiovascular disease; CHD = coronary heart disease; CVD = cardiovascular disease; HF = heart failure.

a *P* < 0.05,

b *P* < 0.01,

c *P* < 0.001.

**Figure 1:**
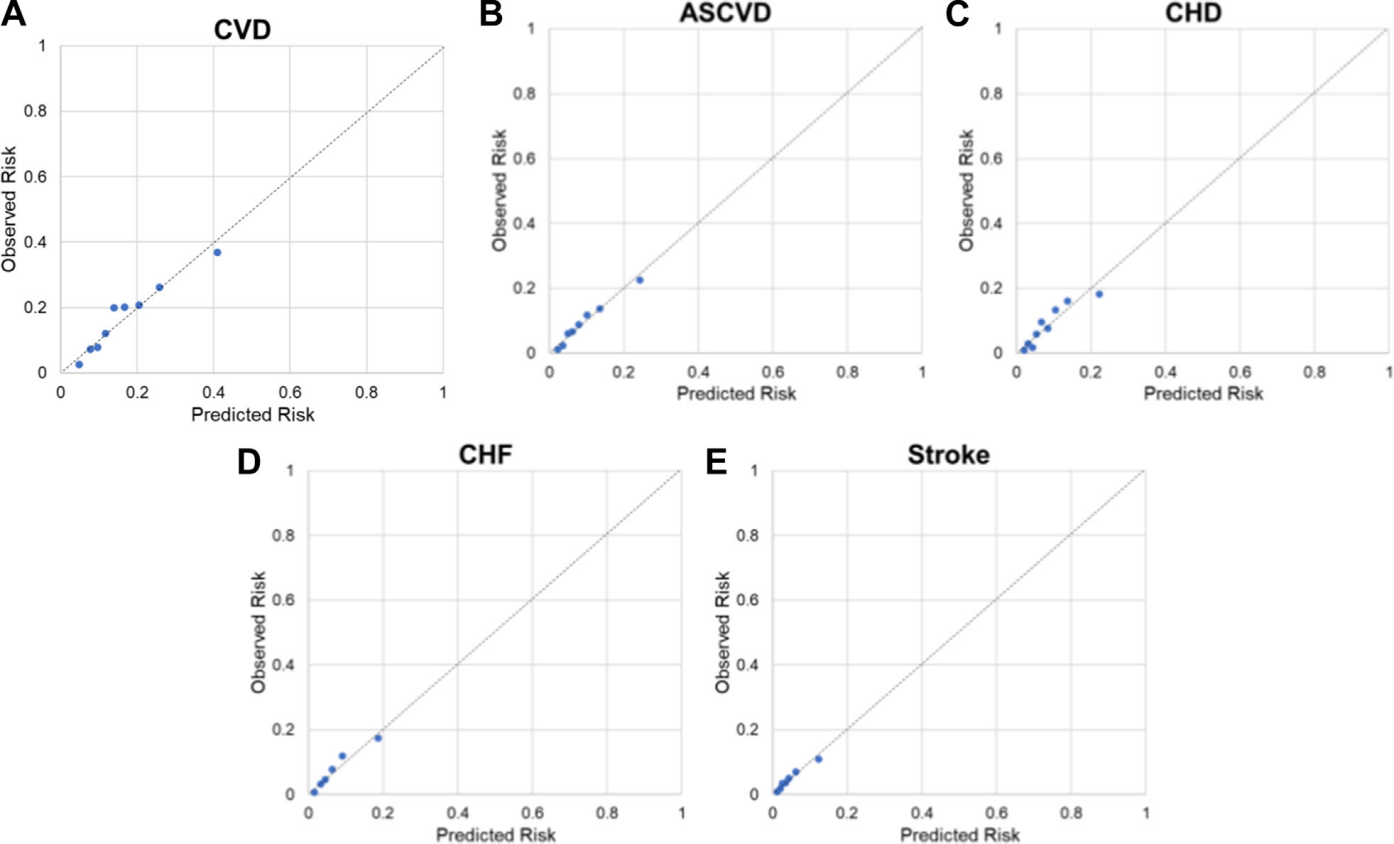
**Internal Calibration Plots for Each Endpoint** (A) CVD; (B) ASCVD; (C) CHD; (D) HF; (E) Stroke. Points on the calibration plots represent the cumulative incidence function of observed 10-year versus mean predicted 10-year risk by different risk scores for decile groups of the predicted risk (groups with events <5 are merged with the next decile group). Calibration lines closer to the diagonal line indicate better calibration performance, with calibration slope=1 and intercept=0 being perfect calibration performance.

Figure 1 shows the calibration plot for each endpoint. The calibration slopes were 0.93 for CVD and 0.95, 0.93, 0.98, and 0.89 for ASCVD, CHD, HF, and stroke, respectively; corresponding calibration intercepts were 0.015, 0.004, 0.006, 0.005, and 0.005 for each endpoint. Calibration parameters by sex were also shown in Table 3. The *P* values of Greenwood Nam-D’Agostino calibration test were statistically significant for CVD (*P* < 0.05) and CHD (*P* < 0.01) but not significant for ASCVD, HF, and stroke.

### External validation

External validation was done using the ARIC (N = 1,781) and ACCORDION (N = 1,160) cohorts. Both cohorts only included participants with DM and free of CVD at baseline. ARIC was 46.0% female with mean age of 57.7 ± 5.7 years, 58.9% White and 40.9% Black; ACCORDION 55.0% female with mean age of 63.0 ± 6.0 years, 60.5% White and 20.8% Black (Table 1).

**Figure 2:**
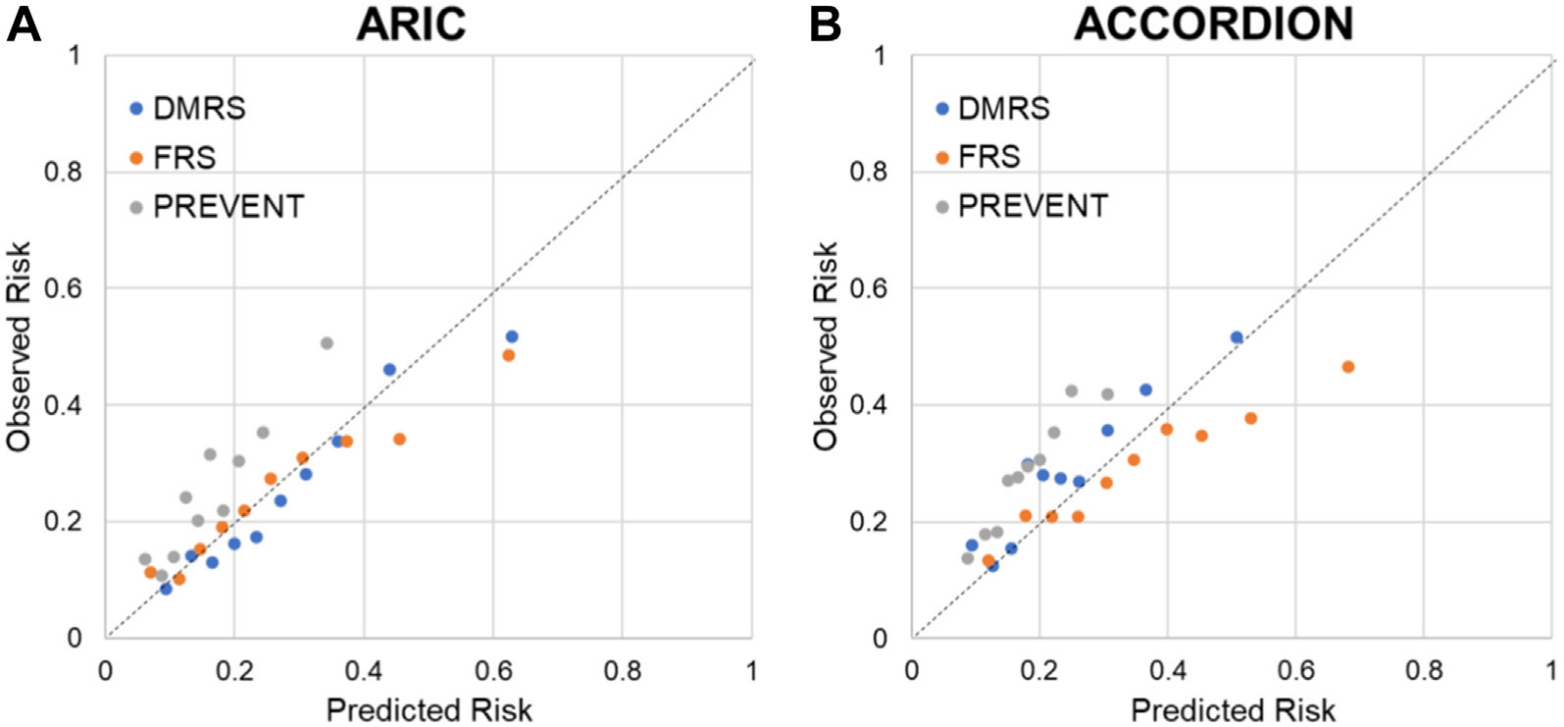
**External Calibration Plots for CVD in Validation Cohorts** (A) ARIC cohort; (B) ACCORDION cohort. Points on the calibration plots represent Kaplan-Meier observed 10-year versus mean predicted 10-year risk by different risk scores for decile groups of the predicted risk. Calibration lines closer to the diagonal line indicate better calibration performance, with calibration slope=1 being perfect calibration performance. Calibration slope <1 indicates overestimation of actual risk. GND Chi-square tests were not statistically significant, indicating excellent external calibration.

In ARIC, the DMRS had C-statistics of 0.69, 0.70, 0.67, 0.75, and 0.67 for CVD, ASCVD, CHD, HF, and stroke, respectively (Supplemental Table 2). The DMRS had superior discrimination over the PREVENT 10-year risk score (all *P* < 0.05 and *P* < 0.0001 for CVD); the DMRS had superior discrimination over FRS for CVD, CHD, and HF (all *P* < 0.01 for comparison); it also showed higher c-statistics than UKPDS (*P* < 0.05 for stroke event). DMRS performed better for the calibration with calibration slopes closer to 1 (Figure 2, Supplemental Table 2). Calibration slopes of DMRS were 0.89, 0.79, 0.75, 0.84, and 0.59 for CVD, ASCVD, CHD, HF, and stroke; calibration slopes of FRS were 0.69, 0.56, and 2.96 for CVD, CHD, and HF; calibration slopes of PREVENT were 1.38, 1.70, and 1.23 for CVD, ASCVD, and HF; calibration slopes of UKPDS were 0.42 for CHD and 0.10 for stroke. UKPDS not only severely overestimated risk but also showed poor discrimination.

ACCORDION showed relatively less discrimination compared to ARIC, with C-statistics of 0.63, 0.65, 0.61, 0.73, and 0.61 for CVD, ASCVD, CHD, HF, and stroke, respectively; calibration performance of DMRS were comparable to that in ARIC: calibration slopes were 0.95, 0.79, 0.71, 0.95, and 0.25 for CVD, ASCVD, CHD, CHF, and stroke, consistently better than FRS, PCE, PREVENT, and UKPDS.

### Application of the DMRS

We used the following individual case study to demonstrate the application of the DMRS: a 50-year-old female diagnosed with DM 5 years ago, current smoker without family history of CVD, on HTN and DM medication, with other risk factors as shown in Supplemental Table 3. Based on her data and the parameters in the risk score equations, her 10-year CVD risk was 17.7%, and 12.3%, 6.6%, 3.2%, and 4.2% for ASCVD, CHD, HF, and stroke events, respectively. With key modifiable risk factors controlled or within normal value (smoking cessation, HbA1c = 7.0%; SBP = 120 mm Hg, total cholesterol = 100 mg/dL), her potential optimal 10-year risks would be 13.8%, 9.2%, 5.5%, 2.6%, and 2.7% for CVD, ASCVD, CHD, HF, and stroke events. We further developed an R-code–based App to implement the DMRS (Supplemental Figure, Supplemental Appendix).

## Discussion

In the current study, we developed a set of DM-specific risk scores for 10-year CVD risk and its components that are uniquely derived from patients with DM from pooled, contemporary, large U.S. community-based cohorts. Age, sex, HbA1c, serum creatinine, SBP, DM medication, and current smoking were the most important predictors of all endpoints. The DMRS demonstrated good to excellent internal discrimination and calibration. In both observational and experimental settings, the DMRS showed superior external validity compared to conventional risk scores including FRS, PCE, PREVENT, and UKPDS.

In the DMRS model development, traditional risk factors including SBP/DBP, total cholesterol, and HDL-C were found to be predictive for future CVD. We also identified some other risk factors that need more attention in adults with DM but are not typically included in CVD risk scores. Among them, serum creatinine was found even more strongly related to CVD risk than well-known risk factors. Our findings are consistent with others showing increased CVD risk with poorer kidney function.^24^^,^^25^ Existing evidence implies the need for measuring kidney function, including UACR, in estimating CVD risk among DM patients; recent review by Khan et al^26^ also suggested the role of albuminuria in predicting HF, which may help explain the better performance for HF seen in the current study. Our DMRS showed good discrimination and excellent calibration in both males and females and in both black and non-black persons internally. The inclusion of DM-specific risk factors including HbA1c, DM duration, and DM medication further increased the external performance. Of note, the recently introduced PREVENT risk scores for total CVD, ASCVD, and HF are a major advance by incorporating some of these factors particularly relevant to those with DM, including HbA1c, eGFR, and urine albumin/creatinine ratio. A social determinant of health measure, zip code, can also further refine risk estimation.^9^ While the PREVENT risk score was developed from a large number of population studies and electronic health record sources, it was not developed from nor has been validated specifically in a DM patient population.

Accurate CVD risk assessment for patients with DM based on individual risk profiles is essential to guide CVD preventive strategies. Although the whole DM population was previously considered as a homogeneous entity regarding macrovascular risk and defined as a “CHD risk equivalent,” contradictory evidence suggests an overall lower CHD risk among patients with DM than those without DM but with a prior CHD, possibly due to the changing definition of DM, earlier diagnosis, and more aggressive preventive treatment.^3^^,^^6^^,^^27^, ^28^, ^29^ Within those with DM, CVD risk may vary by severity of DM as well as the comorbidities, suggesting the importance of including these factors in CVD risk evaluation.^28^^,^^30^ Based on this need for individualized risk assessment, several CVD risk engines for patients with DM have been previously developed,^31^, ^32^, ^33^, ^34^, ^35^, ^36^, ^37^, ^38^ 3 of which could be used among the U.S. DM population but are not without limitations. One used the ARIC cohort^36^ among White and Black subjects estimating overall CVD but not individual CVD endpoints. The other 2 studies based on ACCORD created risk scoring systems for both microvascular and macrovascular complications^37^ and for HF.^38^ Yet these previous scores do not predict the composite of all CVD which includes HF as an important DM comorbidity as our score has included. The SCORE-2 Diabetes Risk Score (11) incorporated 4 large databases comprising mostly European countries and utilized conventional risk factors, as well as age at diabetes diagnosis, HbA1c, and eGFR.

Our DMRS may be useful for the clinician-patient risk discussion for patients with diabetes to help communicate risks for total CVD as well as its individual components and the need for initiation or intensification of tailored therapeutic approaches (eg, statins and antihypertensives for ASCVD and stroke prevention, antihypertensives and SGLT2 inhibitors for HF prevention), as well as to motivate physicians and patients alike to be more motivated to achieve targets for HbA1c, LDL-C, BP, and other measures if not adequately controlled. Those identified to be at higher risk may also, in particular, be candidates for atherosclerosis screening, such as with coronary artery calcium. Upon estimating total and individual CVD risks, we can further identify the potential influences on these risks, providing guidance to hopefully address clinical inertia to reduce these risks. We previously reported composite risk factor control for HbA1c, LDL-C, and BP in the pooled DM cohort of ARIC, JHS, and MESA to be associated with 50% lower CVD risks, with control of LDL-C to have the most benefit in reducing CVD risk but was less frequently at target than BP and HbA1c.^39^ Our DMRS may be helpful to identify those needing more intensive therapy (eg, high intensity statins in those with ≥20% 10-year risk), providing more precision than recommendations aimed in more arbitrarily determining those needing more intensive treatment such as by the presence of additional risk enhancing factors. Utilizing the risk score to evaluate the potential benefit of preventive treatment may further aid clinicians and patients together to help optimize CVD risk reduction by motivating better evaluation of CVD risks and adherence to treatment.

### Study Strengths and Limitations

Our study had several strengths and limitations. Our risk score used 5 major cohorts representing major ethnic groups in the U.S. to develop DM-specific risk scores for macrovascular complications, thus providing greater generalizability than use of only a few cohorts. Our primary CVD endpoint included HF, which is among the most important clinical manifestations of CVD among DM patients.^40^ However, due to the limited number of available variables across our pooled cohorts, some potential predictors, including subclinical atherosclerosis measures, such as coronary artery calcium which has been demonstrated to effectively stratify risk among persons with diabetes,^41^ or novel biomarkers were not able to be examined as candidate variables. However, our focus was to include measures typically available at office visits to optimize clinical utility. Further, we relied on use of baseline risk factor information which does not reflect further changes in risk factors, which may worsen with time, or improve from treatment. Further efforts should be made using dynamic risk profiles to improve risk prediction. We did not distinguish between those with type 1 and type 2 DM in the derivation cohort as some studies did not specifically have this information. Peripheral vascular disease was not adjudicated in JHS and was not included as part of our composite outcome. Moreover, while our risk scores were designed for prediction of CVD outcomes over 10 years, prediction over a longer period, for example, 30 years, has been of recent interest, including with the PREVENT risk scores. Finally, our scores did not and were not designed to predict microvascular complications which should be considered in future research.

## Conclusions

We created 10-year risk prediction scores for CVD, ASCVD, and individual CVD components (CHD, HF, and stroke) specifically for U.S. adults aged 40 to 79 with DM. Our scores had good to excellent internal prediction performance and superior external validation than FRS, PCE, and UKPDS. Use of these scores to identify DM-associated CVD risks based on one’s specific risk profiles may further guide CVD risk factor management for those with DM.

Perspectives **CLINICAL COMPETENCIES:** Cardiovascular disease risk varies greatly in persons with diabetes. A diabetes risk score can help identify those at highest risk needing more intensive treatment.

**TRANSLATIONAL OUTLOOK:** A diabetes risk score based on diverse U.S. population cohorts may be useful for more accurate assessment of cardiovascular risks in persons with diabetes.

## Funding support and author disclosure

Dr Wong has received research funding not related to the current study from Amgen, Novartis, Regeneron, and Novo Nordisk and is a consultant for Amgen, Novartis, Ionis, and Heart Lung. Dr Selvin was supported by 10.13039/100000002NIH/10.13039/100000062NIDDK grants K24 DK106414 and R01 DK089174. The ARIC study is funded by the 10.13039/100000050National Heart, Lung, and Blood Institute under current Contract nos. (HHSN268201700001I, HHSN268201700002I, HHSN268201700003I, HHSN268201700004I, HHSN268201700006I). CARDIA is supported by contracts HHSN268201800003I, HHSN268201800004I, HHSN268201800005I, HHSN268201800006I, and HHSN268201800007I from the 10.13039/100000050National Heart, Lung, and Blood Institute (NHLBI). The Framingham Heart Study is funded by the 10.13039/100000050National Heart, Lung, and Blood Institute under Contract No. 75N92019D00031. JHS (Jackson Heart Study) is supported and conducted in collaboration with 10.13039/100006989Jackson State University (HHSN268201800013I), Tougaloo College (HHSN268201800014I), the 10.13039/100018098Mississippi State Department of Health (HHSN268201800015I/HHSN26800001) and the University of Mississippi Medical Center (HHSN268201800010I, HHSN268201800011I and HHSN268201800012I) contracts from the 10.13039/100000050National Heart, Lung, and Blood Institute (NHLBI) and the National Institute for Minority Health and Health Disparities (NIMHD). The original MESA study was supported by contracts HHSN268201500003I, N01-HC-95159, N01-HC-95160, N01-HC-95161, UL1-TR-000040, N01-HC-95162, UL1-TR-001079, N01-HC-95163, N01-HC-95164, N01-HC-95165, UL1-TR-001420, N01-HC-95166, N01-HC-95167, N01-HC-95168 and N01-HC-95169 from the 10.13039/100000050National Heart, Lung, and Blood Institute, and by grants UL1-TR-000040, UL1-TR-001079, and UL1-TR-001420 from 10.13039/100006108NCATS. The authors have reported that they have no relationships relevant to the contents of this paper to disclose.

## References

[1] Expert Panel on Detection, Evaluation, and Treatment of High Blood Cholesterol in Adults (2001). Executive summary of the third report of the national cholesterol education program (NCEP) expert panel on detection, evaluation, and treatment of high blood cholesterol in adults (adult treatment panel III). JAMA.

[2] Haffner S.M., Lehto S., Rönnemaa T., Pyörälä K., Laakso M. (1998). Mortality from coronary heart disease in subjects with type 2 diabetes and in nondiabetic subjects with and without prior myocardial infarction. N Engl J Med.

[3] Rana J.S., Liu J.Y., Moffet H.H., Jaffe M., Karter A.J. (2016). Diabetes and prior coronary heart disease are not necessarily risk equivalent for future coronary heart disease events. J Gen Intern Med.

[4] Wong N.D., Glovaci D., Wong K. (2012). Global cardiovascular disease risk assessment in United States adults with diabetes. Diabetes Vasc Dis Res.

[5] Malik S., Budoff M.J., Katz R. (2011). Impact of subclinical atherosclerosis on cardiovascular disease events in individuals with metabolic syndrome and diabetes: the multi-ethnic study of atherosclerosis. Diabetes Care.

[6] Zhao Y., Malik S., Budoff M.J. (2021). Identification and predictors for cardiovascular disease risk equivalents among adults with diabetes. Diabetes Care.

[7] D’Agostino R.B., Vasan R.S., Pencina M.J. (2008). General cardiovascular risk profile for use in primary care the Framingham Heart Study. Circulation.

[8] Goff D.C., Lloyd-Jones D.M., Bennett G. (2014). 2013 ACC/AHA guideline on the assessment of cardiovascular risk: a report of the American College of cardiology/American heart association task force on practice guidelines. J Am Coll Cardiol.

[9] Khan S.S., Coresh J., Pencina M.J. (2023). American heart association. Novel prediction equations for absolute risk assessment of total cardiovascular disease incorporating cardiovascular-kidney-metabolic health: a scientific statement from the American heart association. Circulation.

[10] Stevens R.J., Kothari V., Adler A.I., Stratton I.M., United Kingdom Prospective Diabetes Study (UKPDS) Group (2001). The UKPDS risk engine: a model for the risk of coronary heart disease in Type II diabetes (UKPDS 56). Clin Sci (Lond).

[11] SCORE2-Diabetes Working Group and the ESC Cardiovascular Risk Collaboration (2023). SCORE2-Diabetes: 10-year cardiovascular risk estimation in type 2 diabetes in Europe. Eur Heart J.

[12] Rana J.S., Tabada G.H., Solomon M.D. (2016). Accuracy of the atherosclerotic cardiovascular risk equation in a large contemporary, multiethnic population. J Am Coll Cardiol.

[13] Tao L., Wilson E.C., Griffin S.J., Simmons R.K., ADDITION-Europe study team (2013). Performance of the UKPDS outcomes model for prediction of myocardial infarction and stroke in the ADDITION-Europe trial cohort. Value Health.

[14] Friedman G.D., Cutter G.R., Donahue R.P. (1988). CARDIA: study design, recruitment, and some characteristics of the examined subjects. J Clin Epidemiol.

[15] Feinleib M., Kannel W.B., Garrison R.J., McNamara P.M., Castelli W.P. (1975). The Framingham offspring study. Design and preliminary data. Prev Med.

[16] Carpenter M.A., Crow R., Steffes M. (2004). Laboratory, reading center, and coordinating center data management methods in the Jackson Heart Study. Am J Med Sci.

[17] Bild D.E., Bluemke D.A., Burke G.L. (2002). Multi-ethnic study of atherosclerosis: objectives and design. Am J Epidemiol.

[18] The ARIC investigators (1989). The atherosclerosis risk in Communities (ARIC) study: design and objectives. Am J Epidemiol.

[19] ACCORD Study Group (2016). Nine-year effects of 3.7 years of intensive glycemic control on cardiovascular outcomes. Diabetes Care.

[20] Simon N., Friedman J., Hastie T., Tibshirani R. (2011). Regularization paths for Cox’s proportional hazards model via coordinate descent. J Stat Software.

[21] Wilson P.W., D’Agostino R.B., Levy D., Belanger A.M., Silbershatz H., Kannel W.B. (1998). Prediction of coronary heart disease using risk factor categories. Circulation.

[22] Kannel W.B., D'Agostino R.B., Silbershatz H., Belanger A.J., Wilson P.W., Levy D. (1999). Profile for estimating risk of heart failure. Arch Intern Med.

[23] Kothari V., Stevens R.J., Adler A.I. (2002). Ukpds 60: risk of stroke in type 2 diabetes estimated by the UK Prospective Diabetes Study risk engine. Stroke.

[24] Norris K.C., Smoyer K.E., Rolland C., Van der Vaart J., Grubb E.B. (2018). Albuminuria, serum creatinine, and estimated glomerular filtration rate as predictors of cardio-renal outcomes in patients with type 2 diabetes mellitus and kidney disease: a systematic literature review. BMC Nephrol.

[25] Svensson M.K., Cederholm J., Eliasson B., Zethelius B., Gudbjörnsdottir S. (2013). Albuminuria and renal function as predictors of cardiovascular events and mortality in a general population of patients with type 2 diabetes: a nationwide observational study from the Swedish National Diabetes Register. Diabetes Vasc Dis Res.

[26] Khan M.S., Shahid I., Anker S.D. (2023). Albuminuria and heart failure: JACC state-of-the-art review. J Am Coll Cardiol.

[27] Cheng Y.J., Imperatore G., Geiss L.S. (2018). Trends and disparities in cardiovascular mortality among US adults with and without self-reported diabetes, 1988–2015. Diabetes Care.

[28] Bulugahapitiya U., Siyambalapitiya S., Sithole J., Idris I. (2009). Is diabetes a coronary risk equivalent? Systematic review and meta-analysis. Diabet Med.

[29] Kuusisto J., Laakso M. (2013). Update on type 2 diabetes as a cardiovascular disease risk equivalent. Curr Cardiol Rep.

[30] Howard B.V., Best L.G., Galloway J.M. (2006). Coronary heart disease risk equivalence in diabetes depends on concomitant risk factors. Diabetes Care.

[31] Zhao Y., Wong N.D. (2018). The evolving cardiovascular disease risk scores for persons with diabetes mellitus. Curr Cardiol Rep.

[32] Yeboah J., Erbel R., Delaney J.C. (2014). Development of a new diabetes risk prediction tool for incident coronary heart disease events: the Multi-Ethnic Study of Atherosclerosis and the Heinz Nixdorf Recall Study. Atherosclerosis.

[33] Cederholm J., Eeg-Olofsson K., Eliasson B. (2008). Risk prediction of cardiovascular disease in type 2 diabetes: a risk equation from the Swedish National Diabetes Register. Diabetes Care.

[34] Donnan P.T., Donnelly L., New J.P., Morris A.D. (2006). Derivation and validation of a prediction score for major coronary heart disease events in a U.K. type 2 diabetic population. Diabetes Care.

[35] Yang X., So W.Y., Kong A.P. (2008). Development and validation of a total coronary heart disease risk score in type 2 diabetes mellitus. Am J Cardiol.

[36] Parrinello C.M., Matsushita K., Woodward M., Wagenknecht L.E., Coresh J., Selvin E. (2016). Risk prediction of major complications in individuals with diabetes: the Atherosclerosis Risk in Communities Study. Diabetes Obes Metabol.

[37] Basu S., Sussman J.B., Berkowitz S.A., Hayward R.A., Yudkin J.S. (2017). Development and validation of Risk Equations for Complications of type 2 Diabetes (RECODe) using individual participant data from randomised trials. Lancet Diabetes Endocrinol.

[38] Segar M.W., Vaduganathan M., Patel K.V. (2019). Machine learning to predict the risk of incident heart failure hospitalization among patients with diabetes: the WATCH-DM risk score. Diabetes Care.

[39] Wong N.D., Zhao Y., Patel R. (2016). Cardiovascular risk factor targets and cardiovascular disease event risk in diabetes: a pooling project of the atherosclerosis risk in Communities study, multi-ethnic study of atherosclerosis, and Jackson heart study. Diabetes Care.

[40] Shah A.D., Langenberg C., Rapsomaniki E. (2015). Type 2 diabetes and incidence of cardiovascular diseases: a cohort study in 1.9 million people. Lancet Diabetes Endocrinol.

[41] Malik S., Zhao Y., Budoff M. (2017). Coronary artery calcium score for long-term risk classification in individuals with type 2 diabetes and metabolic syndrome from the multi-ethnic study of atherosclerosis. JAMA Cardiol.

