# Supplementary material for: United States Pooled Cohort Cardiovascular Disease Risk Scores in Adults With Diabetes Mellitus
Source: JACC Adv. 2024 Dec 13;4(1):101448. doi: 10.1016/j.jacadv.2024.101448 (PMC11699612; doi:10.1016/j.jacadv.2024.101448)
Supplement: Supplemental data [file mmc1.docx]

**Supplemental Table 1. Baseline characteristics of individual cohorts**

|  | **CARDIA**  **N=349** | **FHS Offspring**  **N=439** | **JHS**  **N=478** | **MESA**  **N=950** |
| --- | --- | --- | --- | --- |
| Age, years | 46.3 ± 3.2 | 63.3 ± 8.3 | 54.8 ± 8.9 | 64.4 ± 8.7 |
| Male | 136 (38.9%) | 196 (49.4%) | 151 (31.8%) | 487 (51.2%) |
| Race groups |  |  |  |  |
| White | 143 (40.9%) | 397 (100%) | 0 (0%) | 203 (21.3%) |
| Black | 207 (59.1%) | 0 (0%) | 475 (100%) | 350 (36.8%) |
| Other races | 0 (0%) | 0 (0%) | 0 (0%) | 399 (41.9%) |
| Above high school education | 249 (71.1%) | 219 (55.2%) | 302 (63.6%) | 507 (53.3%) |
| Current smokers | 67 (19.1%) | 63 (15.9%) | 58 (12.2%) | 103 (10.8%) |
| Alcohol consumption | 246 (70.3%) | 201 (50.6%) | 198 (41.7%) | 355 (37.3%) |
| Family history of CVD | 95 (27.1%) | 87 (21.9%) | 246 (51.8%) | 279 (29.3%) |
| Systolic Blood Pressure, mmHg | 119.3 ± 16.2 | 132.9 ± 18.7 | 129.9 ± 16.5 | 130.0 ± 21.6 |
| Diastolic Blood Pressure, mmHg | 75.3 ± 11.4 | 74.5 ± 9.8 | 76.7 ± 8.4 | 70.8 ± 10.5 |
| Body Mass Index, kg/m^2^ | 34.9 ± 9.9 | 31.0 ± 6.5 | 34.9 ± 7.4 | 31.0 ± 6.2 |
| Waist circumstances, cm | 103.4 ± 17.7 | 108.1 ± 15.7 | 108.9 ± 16 | 105.1 ± 15.1 |
| Total cholesterol, mg/dL | 183.4 ± 40.6 | 195.8 ± 37.7 | 198.5 ± 42.7 | 183.8 ± 36.8 |
| HDL-C, mg/dL | 48.8 ± 16.1 | 47.4 ± 15.6 | 48.7 ± 13.8 | 47.1 ± 13.2 |
| LDL-C, mg/dL | 108.1 ± 36.5 | 115.4 ± 33.5 | 123.2 ± 38.2 | 106.3 ± 32.2 |
| Triglycerides, mg/dL | 138.4 ± 111.2 | 172.1 ± 109.4 | 140.2 ± 119.8 | 158.5 ± 112.5 |
| Serum creatinine, mg/dL | 0.8 ± 0.2 | 0.9 ± 0.2 | 0.9 ± 0.5 | 1.0 ± 0.4 |
| UACR, mg/g | 24.5±60.3 7.5[4.3-17.3] | 62.5±238.6 10.1[4.6-31.8] | 69.2±322.8 11.2[5.5-32.8] | 60.4±217.7 9.7[4.9-27.6] |
| HbA1c, % | 6.7 ± 1.7 | 7 ± 1.5 | 7.6 ± 1.8 | 7.2 ± 1.6 |
| Fasting glucose, mg/dL | 131.5 ± 52.3 | 143.6 ± 50 | 140 ± 57.5 | 142 ± 52.2 |
| DM onset age, years | 38.8 ± 9.7 | 56.4 ± 9.9 | 48.9 ± 10.9 | 59.4 ± 10.7 |
| Heart rate, bpm | 80.5 ± 12.6 | 68.7 ± 12.3 | 69.3 ± 11.6 | 68.2 ± 10.6 |
| Atrial fibrillation | 1 (0.3%) | 15 (3.8%) | 0 (0%) | 0 (0%) |
| Lipid-lowering medication | 130 (7.3%) | 83 (23.7%) | 113 (28.5%) | 95 (20.0%) |
| Hypertension medication | 856 (48.1%) | 148 (42.3%) | 216 (54.4%) | 353 (74.3%) |
| Hypoglycemic medication | 569 (31.9%) | 168 (48.0%) | 157 (39.5%) | 291 (61.3%) |
| Events during 10-year follow-up | | | | |
| CVD | 21 (6%) | 80 (20.2%) | 66 (13.9%) | 172 (18.1%) |
| ASCVD | 19 (5.4%) | 51 (12.8%) | 36 (7.6%) | 91 (9.6%) |
| CHD | 10 (2.9%) | 39 (9.8%) | 29 (6.1%) | 105 (11.0%) |
| HF | 3 (0.9%) | 24 (6.0%) | 29 (6.1%) | 67 (7.0%) |
| Stroke | 11 (3.1%) | 22 (5.5%) | 19 (4.0%) | 35 (3.7%) |
| Continuous variables were presented as mean ± SD (and median [IQR] with skewed distribution); categorical variables are presented as frequency (%).  Percentages of missing values were less than 7% for all variables | | | | |

**Supplemental Table 2. External Validation of Diabetes Mellitus Risk Score (DMRS) in ARIC and ACCORDION (Standard Treatment)**

|  |  | **ARIC**  **N=1,781** | | **ACCORDION**  **N=1,660** | |
| --- | --- | --- | --- | --- | --- |
|  |  | **Discrimination** | **Calibration** | **Discrimination** | **Calibration** |
| **CVD** | DMRS | 0.69 | 0.89/0.002/19.46* | 0.63 | 0.95/0.054/21.82† |
|  | FRS | 0.66† | 0.69/0.064/28.20‡ | 0.61 | 0.57/0.088/351.63‡ |
|  | PREVENT | 0.65‡ | 1.38/0.024/85.41‡ | 0.61 | 1.46/0.020/79.37‡ |
| **ASCVD** | DMRS | 0.70 | 0.79/0.009/24.34† | 0.65 | 0.79/0.038/11.43 |
|  | PCE | 0.68 | 0.72/0.027/18/32* | 0.63 | 0.45/0.042/128.41‡ |
|  | PREVENT | 0.67* | 1.70/-0.019/46.36‡ | 0.64 | 2.23/0.037/226.26‡ |
| **CHD** | DMRS | 0.67 | 0.75/0.019/15.69* | 0.61 | 0.71/0.061/17.41* |
|  | FRS | 0.60‡ | 0.56/0.107/125.45‡ | 0.56*** | 0.39/0.120/97.07‡ |
|  | UKPDS | 0.66 | 0.42/0.069/130.33‡ | 0.62 | 0.24/0.067/616.14‡ |
| **HF** | DMRS | 0.75 | 0.84/-0.009/46.52‡ | 0.73 | 0.95/-0.014/12.31* |
|  | FRS | 0.63‡ | 2.96/0.044/153.91‡ | 0.58** | 0.91/0.036/22.43‡ |
|  | PREVENT | 0.70* | 1.23/-0.003/12.40 | 0.70 | 0.72/-0.021/84.05‡ |
| **Stroke** | DMRS | 0.67 | 0.59/0.018/23.99‡ | 0.61 | 0.25/0.022/74.00‡ |
|  | UKPDS | 0.62† | 0.10/0.056/497.87‡ | 0.57 | 0.02/0.035/4559.42‡ |
| 1. Discrimination is measured by Harrel’s C-statistics; p value is provided by comparing DMRS vs. other risk scores. 2. Calibration is measured by calibration slope/calibration intercept/ GND chi2; p value is provided for GND test. 3. *p<0.05, †p<0.01, ‡p<0.001 | | | | | |

**Supplemental Table 3. Example of Diabetes Mellitus Risk Score Estimation of Cardiovascular Disease Risk in a 50 Year-old White Female**

|  | | Individual X | Beta*X_individual_ | Beta*X_control_^a^ |
| --- | --- | --- | --- | --- |
| Age, per 1 year | | 50 | 1.645 | 1.645 |
| Male | | 0 | 0 | 0 |
| Current smoker | | 1 | 0.510 | 0.510 |
| Family history of CVD | | 0 | 0 | 0 |
| SBP, per 1 mmHg | | 142 | 1.201 | 1.100 |
| HbA1c, per 1% | | 8.2 | 0.594 | 0.507 |
| Waist circumference, per 1cm | | 85 | 0 | 0 |
| Total cholesterol, per 1 mg/dL | | 160 | 0.517 | 0.436 |
| ln(HDL-C), per 1 unit | | ln(45) | -1.783 | -1.783 |
| ln(UACR), per 1 unit | | ln(20) | 0.277 | 0.277 |
| ln(serum creatinine), per 1 unit | | ln(0.9) | -0.063 | -0.063 |
| DM duration over 10 years | | 0 | 0 | 0 |
| Taking medication for HTN | | 1 | 0.230 | 0.230 |
| Taking medication for DM | | 1 | 0.419 | 0.419 |
| Atrial fibrillation | | 0 | 0 | 0 |
| ΣBeta*X | | / | 3.5474 | 3.278 |
| Other parameters in the equation | ΣBeta*X_Mean_ | 3.1897 | | |
|  | S_10_ | 0.8730 | | |
| Final Score | | / | 17.7% | 13.8% |
| The example was a 50-year female with a DM history of 5 years. She is a current smoker without family history of CVD. Her SBP was 142 mmHg, HbA1c was 8.2%, waist circumference was 85 cm, total cholesterol was 160 mg/dL, HDL-C was 45 mg/dL, UACR was 20 mg/g, serum creatinine was 0.9 mg/dL. She took both HTN and DM medications.  Her 10-year CVD risk is calculated as: $R=1-{(S_{10})}^{e^{(\Sigma beta*X_{Individual}-Beta*X_{Mean})}}$, where $\Sigma beta*X_{individual}$ is the sum of beta coefficient*individual’s predictor values.  a. The X_control_ were the individual X value at controlled level for modifiable risk factors as follows: Non-smoker, HbA1c_control_ = 7.0%; SBP_control_ = 130 mmHg; total cholesterol_control_ = 135 mg/dL, waist circumference_control_ = 102cm for men and 88cm for women. If the original X_individual_ value is lower than controlled value, original X_individual is_ used here. | | | | |

**Supplemental Figure**


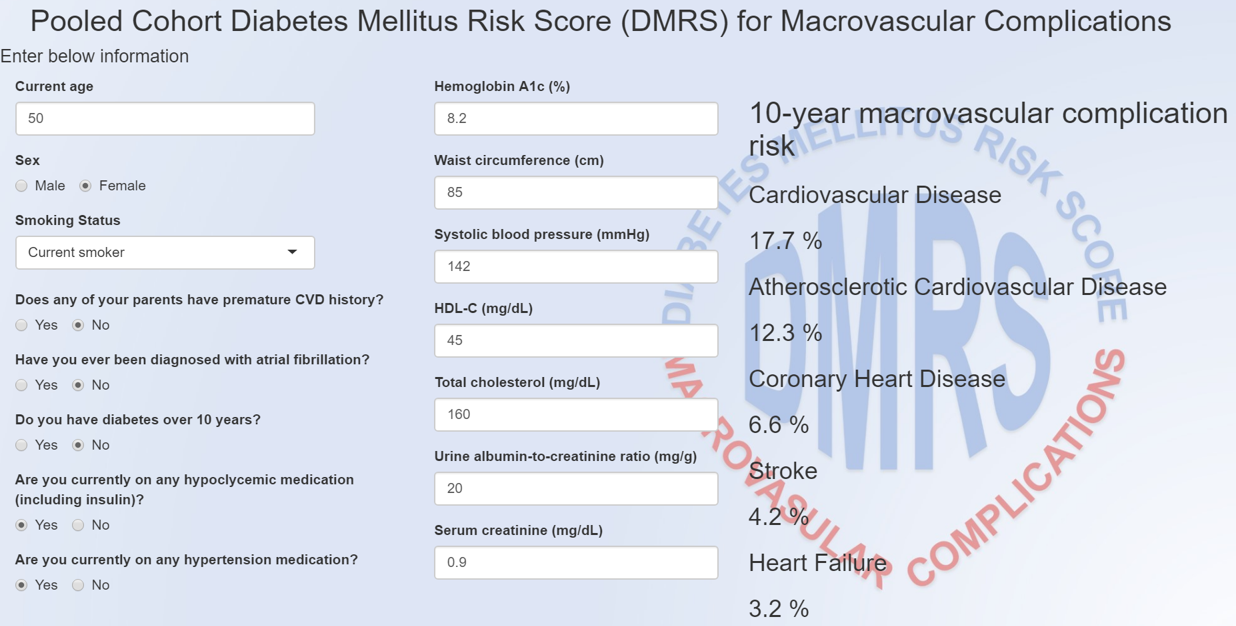


**Appendix: R code to run DM Risk Score calculator**

#Below is the R shiny script to generate the DM Risk Score calculator. R and R studio installation is required.

library(shiny)

library(shinyWidgets)

ui<-fluidPage(

headerPanel(title = h2("Pooled Cohort Diabetes Mellitus Risk Score (DMRS) for Macrovascular Complications")),

fluidRow(

h4("Enter below information"),

column(4,

numericInput("age", "Current age", "50", min=40, max=79),

radioButtons("sex", "Sex",inline = TRUE, list("Male", "Female"), "Female"),

selectInput("smoke", "Smoking Status",

choices = c("Never smoker",

"Previous Smoker",

"Current smoker"),

selected = "Current smoker"),

radioButtons("famhist", "Does any of your parents have premature CVD history?",

inline = TRUE,list("Yes", "No"), "No"),

radioButtons("afib", "Have you ever been diagnosed with atrial fibrillation?", inline = TRUE,list("Yes", "No"), "No"),

radioButtons("dmdura10", "Do you have diabetes over 10 years?",inline = TRUE, list("Yes", "No"), "No"),

radioButtons("dmmed", "Are you currently on any hypoclycemic medication (including insulin)?",inline = TRUE, list("Yes", "No"), "Yes"),

radioButtons("htnmed", "Are you currently on any hypertension medication?", inline = TRUE,list("Yes", "No"), "Yes")

),

column(3,

numericInput("hba1c", "Hemoglobin A1c (%)", "8.2"),

numericInput("waist", "Waist circumference (cm)", "85"),

numericInput("sbp", "Systolic blood pressure (mmHg)", "142"),

numericInput("hdl", "HDL-C (mg/dL)", "45"),

numericInput("chol", "Total cholesterol (mg/dL)", "160"),

numericInput("uacr", "Urine albumin-to-creatinine ratio (mg/g)", "20"),

numericInput("creat", "Serum creatinine (mg/dL)", "0.9")

),

# Main Panel

column(5,

h2("10-year macrovascular complication risk"),

h3("Cardiovascular Disease"),

h3(textOutput("mycvd", inline = TRUE),"%"),

h3("Atherosclerotic Cardiovascular Disease"),

h3(textOutput("myascvd", inline = TRUE),"%"),

h3("Coronary Heart Disease"),

h3(textOutput("mychd", inline = TRUE),"%"),

h3("Stroke"),

h3(textOutput("mystroke", inline = TRUE),"%"),

h3("Heart Failure"),

h3(textOutput("mychf", inline=TRUE),"%")

)

)

)

server <- function(input, output) {

smokeyn<-reactive({if(input$smoke=="Current smoker") 1 else 0})

maleyn<-reactive({if(input$sex=="Male") 1 else 0})

whiteyn<-reactive({if(input$race=="White") 1 else 0})

blackyn<-reactive({if(input$race=="Black") 1 else 0})

otherraceyn<-reactive({if(input$race=="Other race groups") 1 else 0})

famhxyn<-reactive({if(input$famhist=="Yes") 1 else 0})a

dmdurayn<-reactive({if(input$dmdura10=="Yes") 1 else 0})

dmmedyn<-reactive({if(input$dmmed=="Yes") 1 else 0})

htnmedyn<-reactive({if(input$htnmed=="Yes") 1 else 0})

alcoholyn<-reactive({if(input$alcohol=="Yes") 1 else 0})

afibyn<-reactive({if(input$afib=="Yes") 1 else 0})

betaxcvd <- reactive({

0.03289*input$age+

0.30572*maleyn()+

0.51049*smokeyn()+

0*famhxyn()+

0.00846*input$sbp+

0.07244*input$hba1c+

0*input$waist+

0.00323*input$chol+

-0.46827*log(input$hdl)+

0.09247*log(input$uacr)+

0.59951*log(input$creat)+

0.24322*dmdurayn()+

0.23024*htnmedyn()+

0.4187*dmmedyn()+

0.8871*afibyn()

})

dmrscvd<-reactive({round(100*(1-(0.87301^exp(betaxcvd()-3.1897))),1)})

output$mycvd <- dmrscvd

betaxascvd <- reactive({

0.02182*input$age+

0.37574*maleyn()+

0.53093*smokeyn()+

0.45632*famhxyn()+

0.01018*input$sbp+

0.07835*input$hba1c+

-0.01224*input$waist+

0.00356*input$chol+

-0.72228*log(input$hdl)+

0.12305*log(input$uacr)+

0.59436*log(input$creat)+

0*dmdurayn()+

0*htnmedyn()+

0.44462*dmmedyn()+

0.98399*afibyn()

})

dmrsascvd<-reactive({round(100*(1-(0.928724^exp(betaxascvd()-0.66771))),1)})

output$myascvd <- dmrsascvd

betaxchd <- reactive({

0.03965*input$age+

0.56109*maleyn()+

0.5828*smokeyn()+

0*famhxyn()+

0*input$sbp+

0.07171*input$hba1c+

0*input$waist+

0.00391*input$chol+

-0.56944*log(input$hdl)+

0*log(input$uacr)+

0.57837*log(input$creat)+

0.3597*dmdurayn()+

0*htnmedyn()+

0.45244*dmmedyn()+

0*afibyn()

})

dmrschd<-reactive({round(100*(1-(0.933026^exp(betaxchd()-2.02311))),1)})

output$mychd <- dmrschd

betaxstroke <- reactive({

0.0095*input$age+

0.40653*maleyn()+

0*smokeyn()+

0.62337*famhxyn()+

0.01716*input$sbp+

0.12044*input$hba1c+

-0.01324*input$waist+

0.00383*input$chol+

-0.86551*log(input$hdl)+

0.11346*log(input$uacr)+

0*log(input$creat)+

0*dmdurayn()+

0*htnmedyn()+

0.48074*dmmedyn()+

1.36943*afibyn()

})

dmrsstroke<-reactive({round(100*(1-(0.969158^exp(betaxstroke()-0.60494))),1)})

output$mystroke <- dmrsstroke

betaxchf <- reactive({

0.05885*input$age+

0.09877*maleyn()+

0.36235*smokeyn()+

0*famhxyn()+

0*input$sbp+

0.07898*input$hba1c+

0.01333*input$waist+

0.00434*input$chol+

0*log(input$hdl)+

0.21846*log(input$uacr)+

0.66546*log(input$creat)+

0*dmdurayn()+

0.34933*htnmedyn()+

0.50305*dmmedyn()+

1.19112*afibyn()

})

dmrschf<-reactive({round(100*(1-(0.962353^exp(betaxchf()-7.37141))),1)})

output$mychf <- dmrschf

}

shinyApp(ui = ui, server = server)
